# Inappropriate shock caused by P wave oversensing in an entirely subcutaneous ICD

**DOI:** 10.1007/s12471-018-1099-z

**Published:** 2018-03-12

**Authors:** B. A. Mulder, A. H. Maass, Y. Blaauw

**Affiliations:** University Medical Center Groningen, Department of Cardiology, Thoraxcenter, University of Groningen, Groningen, The Netherlands

**Fig. 1a,b:**
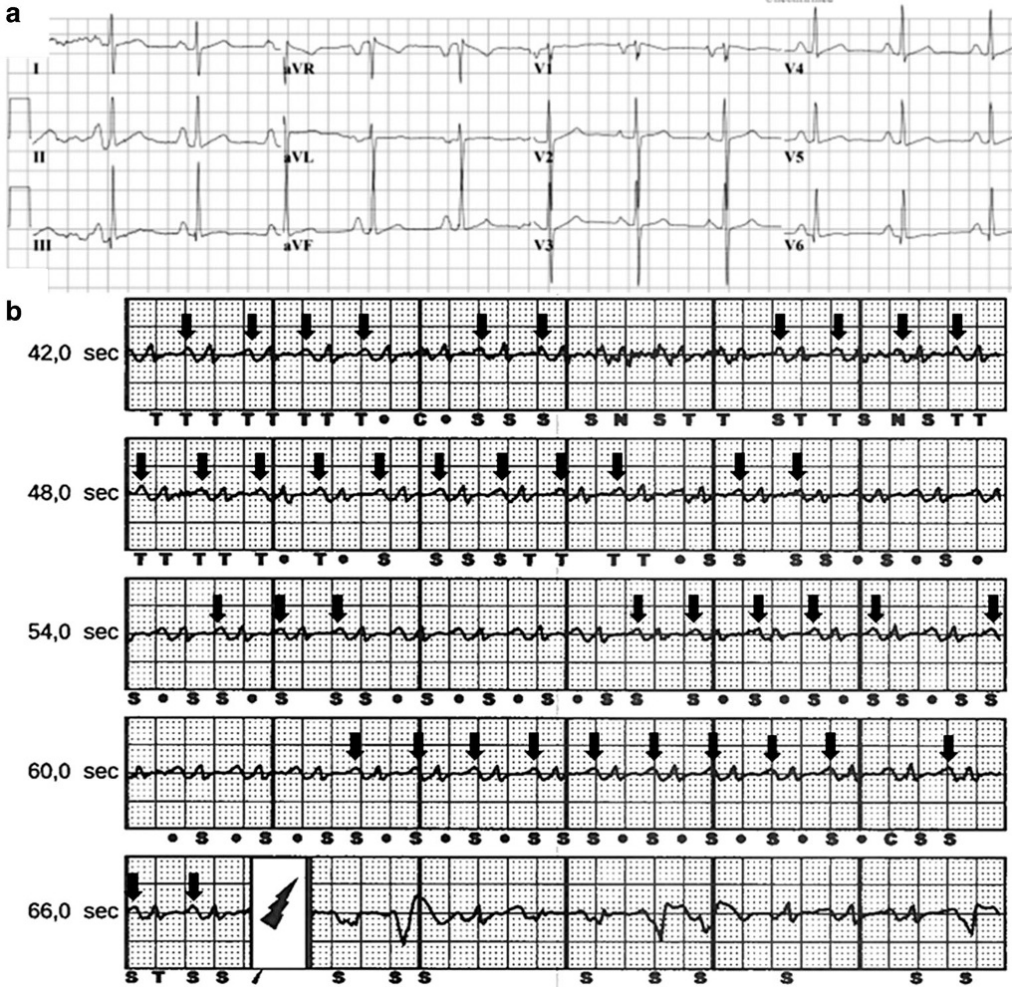
**a** shows electrocardiogram with atrial enlargement, **b** *arrows* indicate all *p*-waves classified as S or T. *S* sensing of an event not classified as tachycardia, *T* sensing of an event classified as tachycardia. *dot* indicates sensing of an unclassifiable event that is discarded, *C* indicated capacitor charging and the *lightning symbol* indicates a shock delivered

A 28-year-old woman with familial hypertrophic cardiomyopathy caused by a mutation in the gene encoding MYH7 presented with an implantable cardioverter defibrillator (ICD) shock to the emergency department. In the past she suffered from non-sustained ventricular tachycardias as well as atrial flutter but she had not experienced an ICD shock before. She cycled home and felt dyspnoeic and shortly thereafter experienced a bang and intense burning sensation retrosternally. ICD interrogation revealed an inappropriate shock because of P wave oversensing. Fig. 1a shows her electrocardiogram with large P waves due to atrial enlargement. Fig. 1b shows inappropriate sensing with detection of both QRS complexes and P waves (arrows indicate sensed P waves). This triggered the ICD to deliver a shock as the calculated frequency exceeded the conditional shock zone. To prevent future shocks, the vector was manually selected and the SMART Pass filter was turned on. During exercise testing appropriate QRS sensing was observed up to 130 beats per minute.

